# Chrysin Inhibits TNFα-Induced TSLP Expression through Downregulation of EGR1 Expression in Keratinocytes

**DOI:** 10.3390/ijms22094350

**Published:** 2021-04-21

**Authors:** Hyunjin Yeo, Young Han Lee, Sung Shin Ahn, Euitaek Jung, Yoongho Lim, Soon Young Shin

**Affiliations:** 1 Department of Biological Sciences, Sanghuh College of Lifesciences, Konkuk University, Seoul 05029, Korea; jini1606@konkuk.ac.kr (H.Y.); yhlee58@konkuk.ac.kr (Y.H.L.); wendy713@konkuk.ac.kr (S.S.A.); mylife4sci@konkuk.ac.kr (E.J.); 2 Division of Bioscience and Biotechnology, BMIC, Konkuk University, Seoul 05029, Korea; yoongho@konkuk.ac.kr

**Keywords:** atopic dermatitis, chrysin, 2,4-dinitrochlorobenzene, early growth response 1, thymic stromal lymphopoietin

## Abstract

Thymic stromal lymphopoietin (TSLP) is an epithelial cell-derived cytokine that acts as a critical mediator in the pathogenesis of atopic dermatitis (AD). Various therapeutic agents that prevent TSLP function can efficiently relieve the clinical symptoms of AD. However, the downregulation of TSLP expression by therapeutic agents remains poorly understood. In this study, we investigated the mode of action of chrysin in TSLP suppression in an AD-like inflammatory environment. We observed that the transcription factor early growth response (EGR1) contributed to the tumor necrosis factor alpha (TNFα)-induced transcription of *TSLP*. Chrysin attenuated TNFα-induced TSLP expression by downregulating EGR1 expression in HaCaT keratinocytes. We also showed that the oral administration of chrysin improved AD-like skin lesions in the ear and neck of BALB/c mice challenged with 2,4-dinitrochlorobenzene. We also showed that chrysin suppressed the expression of EGR1 and TSLP by inhibiting the extracellular signal-regulated kinase (ERK) 1/2 and c-Jun N-terminal kinase (JNK) 1/2 mitogen-activated protein kinase pathways. Collectively, the findings of this study suggest that chrysin improves AD-like skin lesions, at least in part, through the downregulation of the ERK1/2 or JNK1/2-EGR1-TSLP signaling axis in keratinocytes.

## 1. Introduction

Atopic dermatitis (AD), also known as atopic eczema, is a chronic inflammatory skin disease characterized by the development of recurrent eczematous lesions and intense pruritus [1]. The prevalence of AD is constantly growing worldwide over the past 30 years, and nowadays, AD affects about 10% of adults and up to 20% of children [2]. AD is associated with multiple comorbid chronic disorders, such as asthma, allergic rhinitis, respiratory infection, mental disorders, metabolic syndrome, gastrointestinal problems, and cardiovascular disease [3]. A recent cohort study has revealed a variety of clinical forms of AD in adult-onset and childhood-onset types, which may be a crucial factor in determining the appropriate therapeutic medications [4]. AD treatment comprises several types of therapies, such as topical versus systemic application and small molecule inhibitors versus biological agents. Topical therapy includes the application of corticosteroids, antihistamine, and immunosuppressants (e.g., calcineurin inhibitors and phosphodiesterase inhibitors) [5]. Systemic administration comprises immunosuppressant-modulators (e.g., cyclosporine), anti-metabolites (e.g., methotrexate and azathioprine), cytokine signaling inhibitor (e.g., JAK kinase inhibitor), antibiotics, and biological agents (e.g., targeted monoclonal antibodies) [6,7,8,9]. Topical steroids and immunosuppressants have been used as the primary agents; however, their value is limited by local side effects and insufficient efficacy [10]. Oral steroids or immunosuppressants may be used in adults with severe chronic symptoms, but their use is often unsatisfactory due to considerable long-term side effects [11]. Various herbal medicines have been reported to have beneficial effects in the treatment of AD; however, there is not enough evidence to support the use of herbal medicine [10]. Therefore, new systemic therapies with fewer side effects and more efficacious are needed to treat moderate-to-severe chronic AD.

The cause of AD has not been identified in sufficient detail; however, the onset of AD is known to be influenced by genetic and environmental factors, epidermal barrier abnormalities, and impaired cutaneous immune functions [1,12]. In most cases, AD pathogenesis is primarily driven by a milieu of pro-inflammatory cytokines produced by CD4^+^ T helper (Th) lymphocytes, including Th2, Th22, and Th17 cells, as well as pro-inflammatory immune cells, including mast cells, neutrophils, and macrophages/monocytes [13,14,15]. The prominent clinical features of AD include cutaneous inflammation and the chronic itch–scratch–itch cycle, which cause persistent irritation to the skin lesions and impair the skin barrier function. Itching is induced by the stimulation of peripheral sensory neurons by pruritogens. Histamine secreted by mast cells can mediate acute itch in skin inflammation; meanwhile, Th2-associated cytokines, including interleukin (IL)-4, IL-13, and IL-31, directly stimulate sensory neurons [16,17,18].

Thymic stromal lymphopoietin (TSLP) is an IL-17-like cytokine that has been identified and characterized in murine thymic stromal Z210R.1 cells [19,20]. It is produced by various cell types, including stromal cells, epithelial cells, smooth muscle cells, fibroblasts, dendritic cells, mast cells, and epidermal keratinocytes [21]. TSLP promotes the differentiation and growth of B cells and the activation of CD4+/CD8+ T cells and dendritic cells [19,20,22,23,24,25]. In the early stage of AD, keratinocyte-derived TSLP activates dendritic cells to induce the release of various chemokines, which leads to the expansion of the Th2 and Th22 cell populations and induces the release of IL-4, IL-5, IL-13, IL-22, and tumor necrosis factor alpha (TNFα) in large quantities [26]. Ultimately, this results in the persistent activation of Th1 and Th17 cells, impairs epidermal barrier function, accelerates skin inflammation, and promotes the development of AD [27,28]. TSLP also potentially activates mast cells, thus promoting the production of high levels of Th2-like cytokines [29]. TSLP directly stimulates itch sensory neurons independent of Th2 cytokines [30]. Hence, these studies suggest the role of TSLP as a crucial mediator of AD pathogenesis [31] and a potential drug target [32]. Various therapeutic agents that prevent TSLP function can effectively relieve clinical symptoms [33]. TSLP expression is regulated by various cytokines, including pro-inflammatory cytokines, such as TNFα and IL-1, and Th2-related cytokines, such as IL-4, IL-13, and IL-33 [34]. However, the mechanisms underlying TSLP suppression by therapeutic agents remain poorly understood.

**Figure 1:**
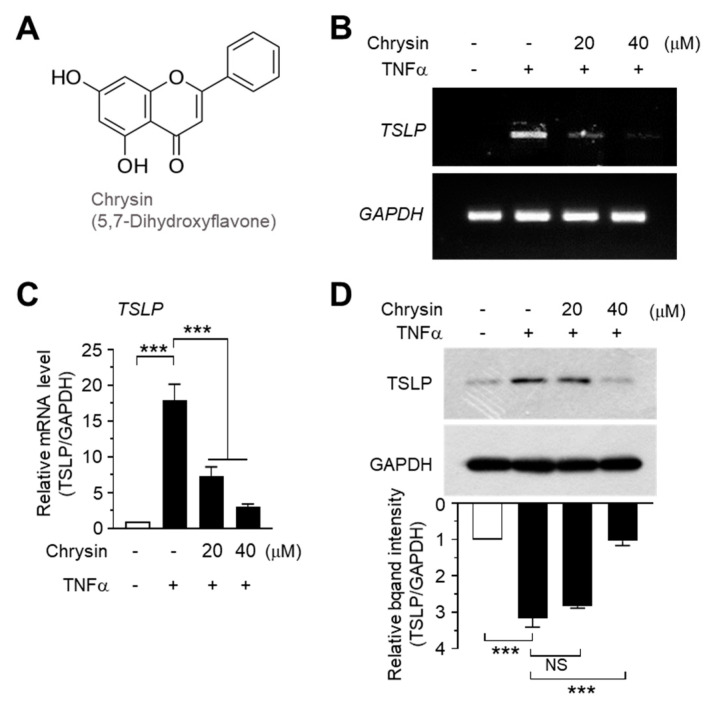
Effect of chrysin on the suppression of TNFα-induced *TSLP* expression. (**A**) Chemical structure of chrysin (5,7-dihydroxyflavone). (**B**) HaCaT cells were pretreated with chrysin (20 and 40 μM) for 30 min before stimulation with 10 ng/mL TNFα. After 12 h, total RNA was isolated, and the levels of *TSLP* mRNA were measured using RT-PCR. *GAPDH* mRNA was used as an internal control. Minus (−), vehicle treatment; Plus (+), TNFα treatment. (**C**) HaCaT cells were treated as in (**B**), and total RNA was isolated. *TSLP* mRNA levels were quantified using quantitative real-time PCR with SYBR Green-based fluorescent probes. The relative expression was normalized to the *GAPDH* mRNA levels. The relative *TSLP* mRNA level in the untreated cells was designated 1. Data are expressed as mean ± SD (*n* = 3); *** *p* < 0.001 by Dunnett’s multiple comparisons test. Minus (−), vehicle treatment; Plus (+), TNFα treatment. (**D**) HaCaT cells were pretreated with chrysin (20 and 40 μM) for 30 min and then stimulated with 10 ng/mL TNFα for 24 h. The quantity of TSLP protein was measured using Western blot analysis. The band intensity corresponding to each TSLP protein was normalized to the GAPDH level using ImageJ v1.52a software. Data are expressed as mean ± SD (*n* = 3). NS, not significant; *** *p* < 0.001 by Dunnett’s multiple comparisons test. Minus (−), vehicle treatment; Plus (+), TNFα treatment.

Chrysin (5,7-dihydroxyflavone, Figure 1A) is a flavonoid found in large quantities in honey, propolis, mushrooms, and carrot. It exhibits multiple pharmacological and therapeutic properties, including neuroprotective, anti-inflammatory, and anticancer properties [35,36]. Notably, chrysin is known to alleviate AD by inhibiting the production of multiple pro-inflammatory cytokines and chemokines [37,38,39]. Choi et al. [37] have demonstrated that chrysin significantly inhibits the production of cytokines, Th2 chemokines, CCL17, and CCL22 by the downregulation of p38 MAPK, NF-κB, and STAT1 in TNFα/IFNγ-stimulated HaCaT keratinocytes. However, despite the beneficial effects of chrysin in AD therapy, the mechanism underlying the suppression of TSLP expression by chrysin remains unclear.

In this study, we attempted to elucidate the role of chrysin in TSLP suppression in keratinocytes. We found that chrysin inhibited TNFα-induced TSLP expression by downregulating mitogen-activated protein kinase (MAPK)-mediated EGR1 expression in HaCaT keratinocytes. In addition, we demonstrated that the oral administration of chrysin suppressed EGR1 and TSLP expression in AD-like skin lesions in BALB/c mice. 

## 2. Results

### 2.1. Chrysin Inhibits TNFα-Induced TSLP Expression in HaCaT Keratinocytes

Previous studies have shown that chrysin alleviates AD-like skin lesions in a mouse model [37] and reverses the NF-κB-mediated inhibition of C-C motif chemokine ligand (CCL) 5 [39]. TSLP plays a key role in AD progression, and TSLP upregulation is considered a hallmark of AD pathogenesis [31,32]. TNFα is a pro-inflammatory cytokine that promotes inflammation by inducing the production of various other inflammatory cytokines and chemokines [40]. TNFα production was enhanced in a mouse model of 2.4-dinitrobenzene (DNCB)-induced contact allergy [41], and TNFα induced TSLP expression in skin keratinocytes [42]. To investigate whether chrysin modulates TSLP expression, we used TNFα as a positive signal to induce TSLP expression. As reported in a previous study [42], the *TSLP* mRNA levels were enhanced upon TNFα stimulation, as shown using reverse transcription (RT)-PCR (Figure 1B). However, chrysin pretreatment abrogated the ability of TNFα to induce *TSLP* mRNA expression. The changes in *TSLP* mRNA levels were measured using quantitative real-time PCR (Q-PCR) with *TSLP-*specific SYBR Green-based fluorescent probes. TNFα increased the *TSLP* mRNA level by 17.9 ± 2.52-fold compared to that in the control; however, upon treatment with 20 and 40 μM chrysin, the *TSLP* mRNA levels decreased by 7.40 ± 1.45- and 2.97 ± 0.397-fold, respectively, compared to the levels in the control (Figure 1C). Chrysin consistently suppressed TNFα-induced TSLP accumulation in a dose-dependent manner (Figure 1D). These data suggest that chrysin inhibits TNFα-induced TSLP expression at the mRNA level.

### 2.2. The Chrysin Response Element Is Located between the −369 and +18 Positions in the TSLP Promoter

**Figure 2:**
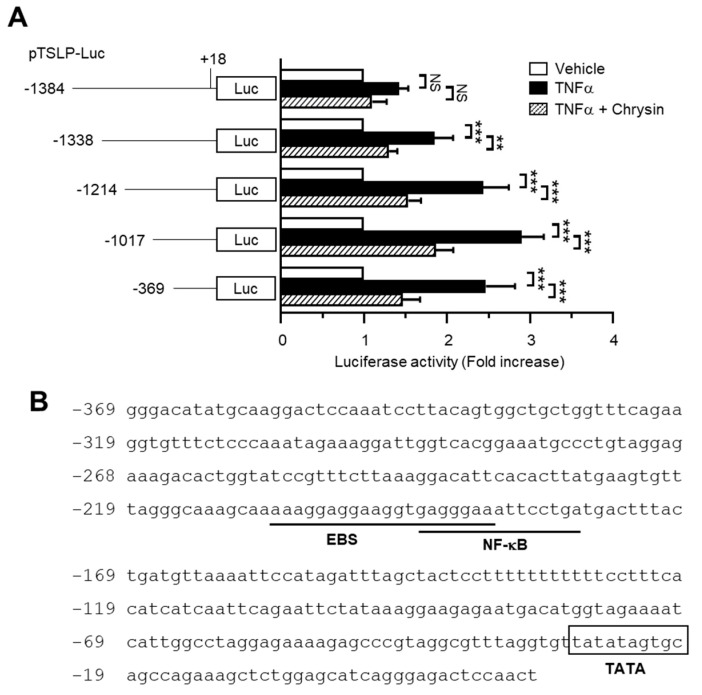
Effect of chrysin on the inhibition of TNFα-induced *TSLP* promoter activity. (**A**) HaCaT cells were transfected with 0.2 µg of a set of 5′-deletion constructs of *TSLP* promoter-reporter plasmids. At 48 h post-transfection, the cells were treated with 10 ng/mL TNFα in the absence or presence of 40 μM chrysin. After 8−12 h, the cells were harvested, and the luciferase reporter activities were measured. The schematic diagram shows the set of deletion constructs of the *TSLP* promoter–reporter plasmid. Data are expressed as mean ± SD (*n* = 3). NS, not significant; ** *p* < 0.01; *** *p* < 0.001 by Sidak’s multiple comparisons test. (**B**) Nucleotide sequence of the 5’-regulatory region of human *TSLP* spanning between the positions −369 and +18. The EGR1-binding sequence and NF-κB sites are underlined. The TATA box (−29/−22) is indicated using the box.

To elucidate the effect of chrysin on the inhibition of TNFα-induced *TSLP* expression, we established a series of *TSLP* promoter deletion constructs: −1384/+18, −1338/+18, −1214/+18, −1017/+18, and −369/+18. These constructs harbored the luciferase reporter gene. Each of these promoter-reporters was transiently transfected into HaCaT cells, and the luciferase activity was measured following TNFα stimulation. As shown in Figure 2A, TNFα-induced *TSLP* promoter–reporter activity was persistently repressed in cells transfected with the shortest construct (−369/+18), suggesting that the chrysin response element is located between the −369 and +18 positions.

To identify the chrysin response elements, we analyzed the transcription factor-binding sites between the −369 and +18 positions using MatInspector program (Genomatix Software, Munich, Gertmany). The nuclear factor kappa B (NF-κB)-binding site was found to overlap with a putative early growth response 1 (EGR1)-binding sequence (EBS) located in the region between positions −206 and −187 (Figure 2B).

### 2.3. Chrysin Inhibits the DNA-Binding Activity of EGR1

**Figure 3:**
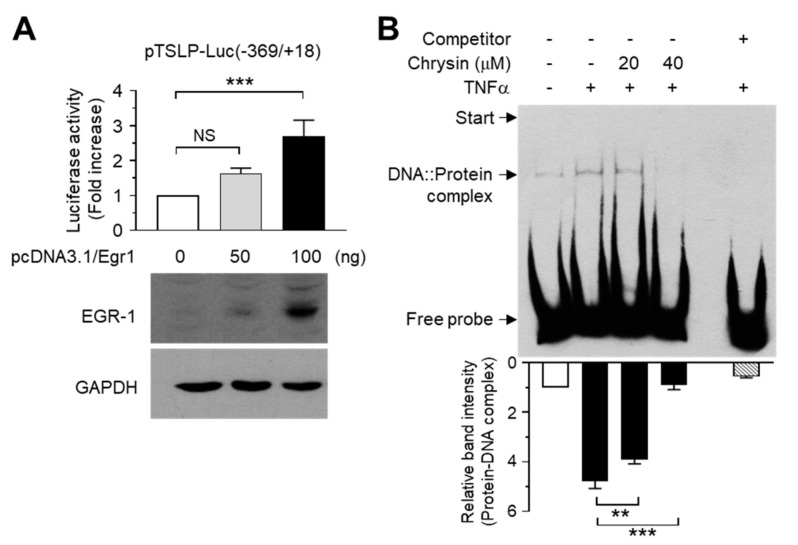
Chrysin inhibits the DNA-binding activity of EGR1. (**A**) HaCaT cells were co-transfected with the pTSLP-Luc(−369/+18) reporter plasmid at increasing concentrations of the EGR1 expression plasmid. After 48 h, the cells were harvested, and the luciferase activities were measured (*top graph*). Bars represent means ± SD (*n* = 3). NS, not significant; *** *p* < 0.001 by Dunnett’s multiple comparisons test. Expression of EGR1 post-transfection was confirmed using Western blotting (*bottom panels*). Glyceraldehyde-3-phosphate dehydrogenase (GAPDH) was used as an internal control. (**B**) HaCaT cells were treated with or without 10 ng/mL TNFα for 1 h in the presence or absence of chrysin (20 and 40 μM). Nuclear extracts (3 μg) were prepared and incubated with a biotinylated *EGR1*-binding oligonucleotide probe (50 fmole) in the absence or presence of an unlabeled competitor (2500 fmole). The samples were separated by electrophoresis in non-denaturing 6% polyacrylamide gels and incubated with streptavidin-conjugated horseradish peroxidase. Protein–DNA complexes were visualized using a Western blotting detection kit (*top panel*). The intensity of the protein–DNA complexes was measured using ImageJ v1.52a software (*bottom graph)*. ** *p* < 0.01; *** *p* < 0.001 by Dunnett’s multiple comparisons test 2.4. Minus (−), vehicle treatment; Plus (+), TNFα or competitor treatment.

Previous studies have shown the role of NF-κB in mediating TNFα-induced TSLP expression in human airway smooth muscle cells [43] and IL-1β-induced TSLP expression in intestinal epithelial cells [44]. The transcription factor EGR1 mediates IL33-induced TSLP expression in keratinocytes [45]. However, the role of EBS in the −369/+18 region of the *TSLP* promoter remains elusive. We focused on the role of EGR1 in chrysin-mediated TSLP suppression. To determine whether EGR1 transactivates the EBS in the −369/+18 construct, we co-transfected the −369/+18 construct and an expression plasmid for EGR1 (pcDNA3.1/Egr1) and measured the luciferase reporter activity. Exogenous EGR1 expression increased the promoter–reporter activity of the −369/+18 construct in a plasmid concentration-dependent manner (Figure 3A), suggesting that the putative EBS in the −369/+18 construct could be a functional *cis-*acting element for EGR1 that participates in TNFα-induced *TSLP* transcription.

To determine whether chrysin affects the binding of EGR1 to the putative EBS in the −369/+18 region, we performed the electrophoretic mobility shift assay (EMSA). Nuclear extracts from HaCaT cells treated with TNFα in the presence or absence of chrysin were incubated with a biotinylated EBS oligonucleotide probe, and the DNA-binding proteins were analyzed using streptavidin-conjugated horseradish peroxidase. Unlabeled EBS competitors were administered at a fifty-fold excess (2.5 pmol) concentration to indicate the specific reaction of the DNA–protein complex formation. Figure 3B shows that TNFα promoted the formation of the DNA–protein complex; however, the concentration of this complex was significantly (*p* < 0.01) reduced upon chrysin pretreatment, suggesting that EGR1 interacts with the putative EBS in the −369/+18 region of the *TSLP* promoter. 

### 2.4. Chrysin Downregulates EGR1 Expression to Inhibit TNFα-Induced TSLP Expression

**Figure 4:**
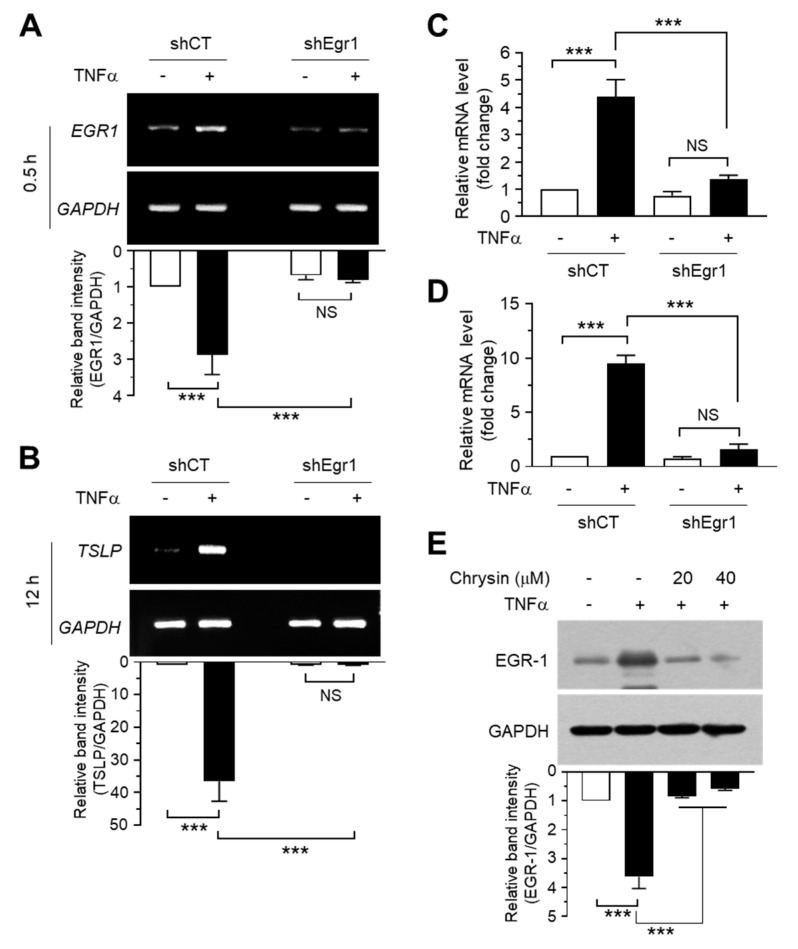
Inhibition of *TSLP* mRNA expression via *EGR1* knockdown and the effect of chrysin on the suppression of TNFα-induced EGR1 expression. (**A,B**) HaCaT transfectants expressing scrambled (shCT) or *EGR1* shRNA (shEgr1) were treated with 10 ng/mL TNFα for 30 min (**A**) or 12 h (**B**). Total RNA was isolated, and *EGR1* (**A**,**C**) or *TSLP* mRNA expression (**B**,**D**) was measured using RT-PCR (**A**,**B**) and quantitative real-time PCR (**C**,**D**). The *GAPDH* mRNA level was measured as an internal control. RT-PCR product intensities were measured using the ImageJ v1.52a software. Data are presented as mean ± SD (*n* = 3). NS, not significant; *** *p* < 0.001 by Sidak’s multiple comparisons test. (**E**) HaCaT cells expressing scrambled (shCT) or short-hairpin *EGR1* shRNA (shEgr1) were incubated with 0.5% serum for 24 h, followed by treatment with 10 ng/mL TNFα for 1 h in the presence or absence of chrysin. The cell lysates were immunoblotted using anti-EGR1 antibodies. Glyceraldehyde-3-phosphate dehydrogenase (GAPDH) was used as an internal control. The band intensity corresponding to each EGR1 protein was normalized to the GAPDH level using ImageJ v1.52a software. *** *p* < 0.001 by Dunnett’s multiple comparisons test. Minus (−), vehicle treatment; Plus (+), TNFα treatment.

To further confirm whether EGR1 is required for TNFα-induced *TSLP* expression, we silenced EGR1 expression by expressing the control scrambled shRNA (shCT) or *EGR1* shRNA (shEgr1) in HaCaT cells. The knockdown of *EGR1* expression was confirmed using RT-PCR (Figure 4A) and Q-PCR (Figure 4C). The *TSLP* mRNA expression-inducing potential of TNFα was significantly (*p* < 0.001) inhibited in HaCaT/shEgr1 cells compared to HaCaT/shCT cells, as revealed by RT-PCR (Figure 4B). The decrease in *TSLP* mRNA levels by shEgr1 expression was quantitated using Q-PCR analysis. TNFα-induced *TSLP* mRNA expression increased 9.50 ± 0.755-fold in HaCaT/shCT cells but only 1.80 ± 0.300-fold in HaCaT/shEgr1 cells (Figure 4D). These data suggest that EGR1 plays a critical role in TNFα-induced *TSLP* transcription.

Then, we determined whether chrysin affects EGR1 expression. Serum-starved HaCaT cells were treated with 10 ng/mL TNFα for 1 h in the presence or absence of chrysin, and the EGR1 levels were measured using immunoblotting. TNFα-induced EGR1 accumulation was significantly (*p* < 0.001) abrogated after chrysin pretreatment (Figure 4E). These results suggested that chrysin downregulated EGR1 expression to suppress *TSLP* transcription. 

### 2.5. Oral Administration of Chrysin Attenuates 2,4-Dinitrochlorobenzene (DNCB)-Induced AD-Like Skin Lesions in BALB/c Mice

**Figure 5:**
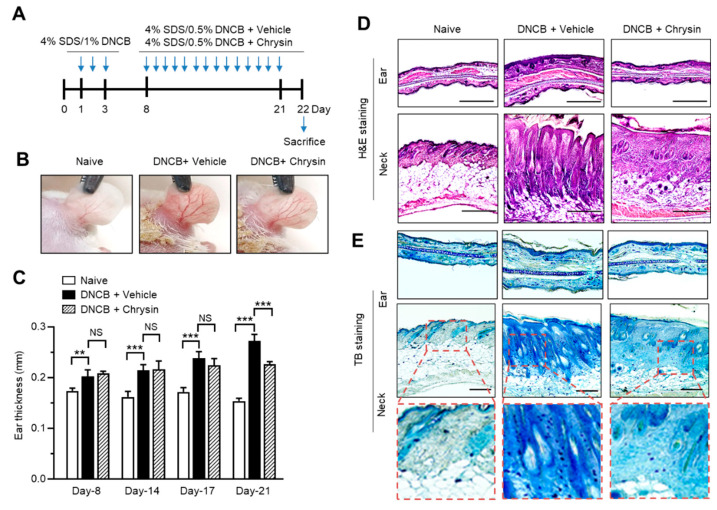
Effect of oral chrysin administration on the attenuation of skin lesions in DNCB-challenged BALB/c mice. (**A**) Illustration of the experimental schedule for the induction of atopic dermatitis-like skin lesions and oral chrysin administration. (**B**) Representative images of the ear and neck skin of BALB/c mice; untreated control (naive), DNCB + vehicle (PBS), and DNCB + chrysin (25 mg/kg). Images were acquired on day 22 immediately after the mice were euthanized. (**C**) Ear thickness was measured using a micro caliper after DNCB challenge. Data are expressed as mean ± SD (*n* = 3). NS, not significant; ** *p* < 0.01; *** *p* < 0.001 by Sidak’s multiple comparisons test. (**D**,**E**) Paraffin-embedded ear and neck skin tissues of BALB/c mice were prepared on day 22, and H&E (**D**) and TB staining (**E**) were performed. The enlarged version of each image is provided in the dotted boxes. Scale bars, 400 μm.

DNCB has been widely used as an inducer of AD-like skin lesions in mouse models [46]. Chrysin was shown to attenuate DNCB-induced skin lesions [37]. To confirm the effect of chrysin on in vivo TSLP suppression, we induced AD-like skin inflammation by topical sensitization with SDS and DNCB (Figure 5A). The ear skin subjected to repeated DNCB applications exhibited typical signs of AD-like skin lesions, such as superficial erosion; however, the signs of DNCB-induced skin erosion were substantially attenuated by the oral administration of chrysin (25 mg/kg) compared to those in the DNCB-challenged group (Figure 5B). Skin edema is a typical sign of skin inflammation in mouse models. We monitored ear swelling by measuring the ear thickness throughout the experimental period of 21 days. DNCB-challenged mice exhibited ear swelling in a time-dependent manner; however, the oral administration of chrysin significantly reduced the ear thickness on day 21 (Figure 5C). Hematoxylin and eosin (H&E) staining of the tissue sections revealed that oral chrysin administration attenuated DNCB-induced epidermal hyperplasia of the ear and neck skin tissues (Figure 5D). DNCB-induced AD-like skin lesions are also characterized by the massive infiltration of various immune cells, including T cells and mast cells, into the inflammatory regions [47]. We studied the effect of chrysin on the inhibition of immune cell infiltration using toluidine blue (TB) staining [48]. DNCB application increased the infiltration of TB-positive cells, whereas the oral administration of chrysin substantially suppressed the DNCB-induced infiltration of TB-positive cells (Figure 5E). These data confirmed the beneficial effect of chrysin on DNCB-induced AD-like skin lesions in a mouse model.

### 2.6. Oral Administration of Chrysin Reduces EGR1 and TSLP Expression in DNCB-Induced Skin Lesions in BALB/c Mice

**Figure 6:**
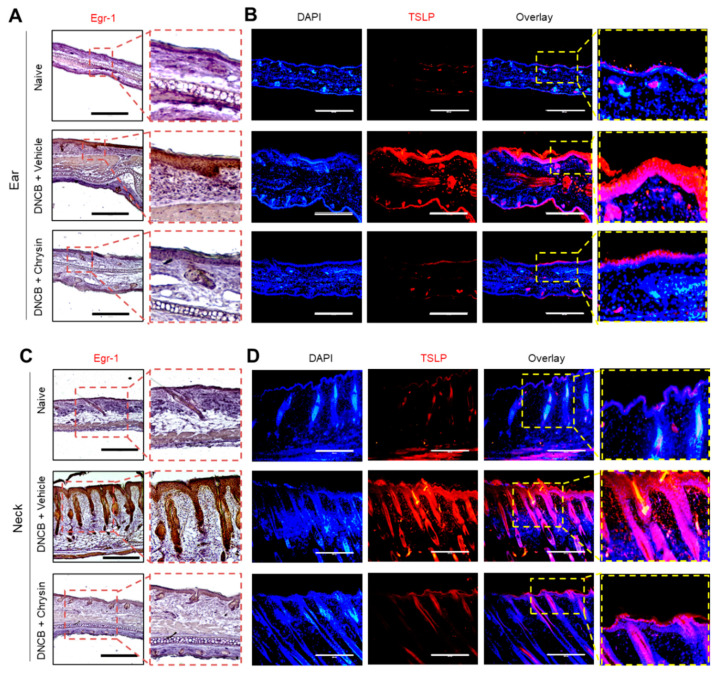
Effect of chrysin on the suppression of EGR1 and TSLP expression in DNCB-challenged BALB/c mice. (**A**,**C**) BALB/c mice either untreated (naive) or treated with DNCB + vehicle (PBS) and DNCB + chrysin. Paraffin-embedded ear (**A**) and neck (**C**) tissue sections were prepared on day 22, and immunohistochemical staining was performed for EGR1. The sections were counterstained with H&E. Scale bars, 400 μm. The areas in the dashed boxes are enlarged in the bottom panels. (**B**,**D**) Paraffin-embedded ear (**B**) and neck (**D**) tissue sections were prepared and subjected to immunofluorescence staining with an anti-TSLP antibody and rhodamine red-X-conjugated secondary antibody (red). The nuclei were counterstained with Hoechst 33258 (blue). Scale bars, 400 μm. The areas in the dashed boxes are enlarged in the panels to the right.

We next evaluated whether the oral administration of chrysin could suppress EGR1 and TSLP expression in AD-like skin lesions in BALB/c mice. Immunohistochemical analysis of the skin sections showed that DNCB increased EGR1-positive staining in the epidermis of the ear (Figure 6A) and neck (Figure 6C). Notably, EGR1-positive staining induced under DNCB challenge was substantially suppressed in response to the oral administration of chrysin. Similarly, immunofluorescence staining showed that the levels of TSLP-positive staining in the epidermis of the ear (Figure 6B) and neck (Figure 6D) reduced upon the oral administration of chrysin. These results support the notion that chrysin inhibits TSLP expression by downregulating EGR1 in inflammatory microenvironments. 

### 2.7. Chrysin Inhibits the MAPK Pathways

**Figure 7:**
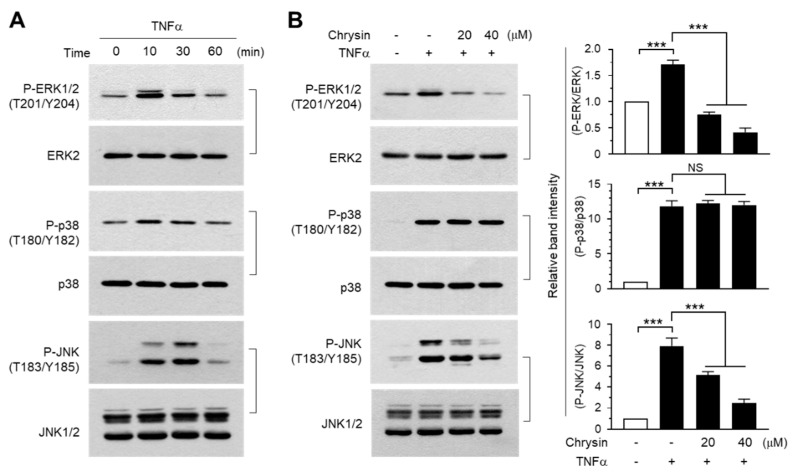
Effect of chrysin on the inhibition of mitogen-activated protein kinases (MAPKs). (**A**) HaCaT cells were treated with 10 ng/mL TNFα for 0–60 min. (**B**) HaCaT cells were treated with 10 ng/mL TNFα for 10 min in the presence or absence of chrysin at different concentrations (20 and 40 μM). Whole-cell lysates were prepared, and Western blotting was performed using phospho-specific and total MAPK protein antibodies. The band intensities of the phosphorylated proteins were normalized relative to those of total proteins, using ImageJ v1.52a software. Data are expressed as mean ± SD (*n* = 3) in the graphs. NS, not significant; *** *p* < 0.001 by Dunnett’s multiple comparisons test. Minus (−), vehicle treatment; Plus (+), TNFα treatment.

We investigated the mode of action of chrysin, which is considered to inhibit EGR1 expression and downregulate TSLP expression. MAPK pathways regulate EGR1 expression in various cell types [49,50,51]. The levels of phosphorylated ERK1/2, JNK1/2, and p38 kinase increased rapidly within 10 min following TNFα treatment, whereas the total quantity of each MAPK protein did not change (Figure 7A). Under these experimental conditions, the effect of chrysin on MAPK phosphorylation was examined. We observed that chrysin significantly (*p* < 0.001 in all cases) inhibited the TNFα-induced phosphorylation of ERK1/2 and JNK1/2, but not of p38 kinase (Figure 7B). These data suggest that while the three major MAPKs are activated by TNFα in HaCaT cells, chrysin selectively inhibits only the ERK1/2 and JNK1/2 MAPK pathways.

### 2.8. MAPK Pathways Are Involved in TNFα-Induced EGR1 and TSLP Expression in HaCaT Keratinocytes

**Figure 8:**
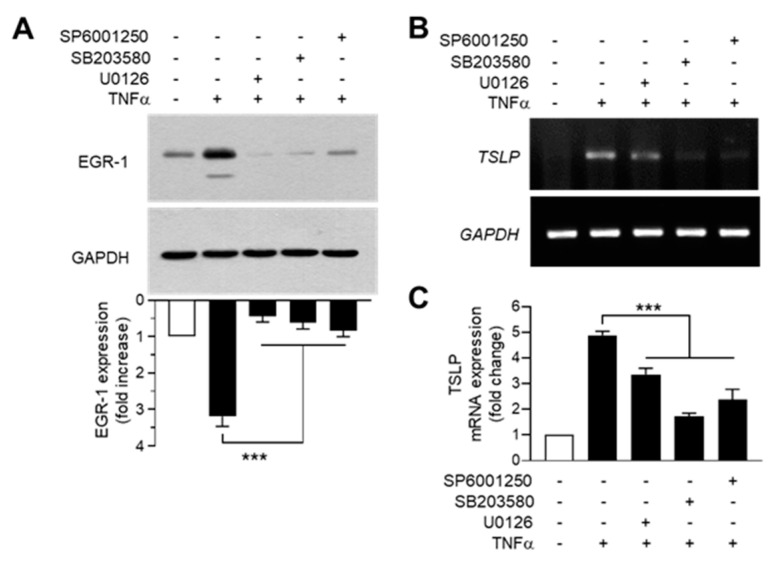
Effect of mitogen-activated protein kinase inhibition on the expression of TNFα-induced EGR1 and *TSLP*. (**A**) HaCaT cells were pretreated with SB203580 (20 µM), U0126 (10 µM), or SP600125 (20 µM) for 30 min, followed by treatment with 10 ng/mL TNFα for 1 h. Whole-cell lysates were prepared, and Western blotting was performed using anti-EGR1 antibodies. Glyceraldehyde-3-phosphate dehydrogenase (GAPDH) was used as an internal control. The band intensity corresponding to EGR1 was normalized to the GAPDH level using the ImageJ v1.52a software. The graphical data show mean ± SD values (*n* = 3). *** *p* < 0.001 using Dunnett’s multiple comparisons test. (**B**,**C**) HaCaT cells were pretreated with SB203580 (20 µM), U0126 (10 µM), or SP600125 (20 µM) for 30 min, followed by treatment with 10 ng/mL TNFα for 12 h. Total RNA was isolated, and the levels of *TSLP* mRNA were measured using RT-PCR (**B**) and quantitative real-time PCR (**C**). *GAPDH* mRNA was used as an internal control. Data are expressed as mean ± SD (*n* = 3). *** *p* < 0.001 by Dunnett’s multiple comparisons test. Minus (−), vehicle treatment; Plus (+), TNFα or inhibitor treatment.

To determine the potential relationship between MAPK activation and TNFα-induced EGR1 expression, we used pharmacological inhibitors of the MAPK pathway. Pretreatment with the MAPK kinase inhibitor U0126, p38 kinase inhibitor SB203580, or JNK inhibitor SP600125 significantly (*p* < 0.001 in all cases) decreased TNFα-induced EGR1 accumulation, as revealed in the Western blot analysis (Figure 8A). Findings from the RT-PCR (Figure 8B) and real-time PCR (Figure 8C) analyses indicated that TNFα-induced *TSLP* mRNA expression was significantly inhibited by all three MAPK inhibitors (*p* < 0.001 in all cases). These data suggest that all three MAPKs mediate TNFα-induced *TSLP* expression via EGR1, but chrysin selectively inhibits only the TNFα-induced ERK1/2 and JNK1/2 pathways to downregulate *TSLP* expression.

## 3. Discussion

Chrysin has a pharmacological property that helps alleviate the clinical symptoms of AD by inhibiting the secretion of pro-inflammatory cytokines and chemokines [37,38,39]. TNFα is a major pro-inflammatory cytokine that is released from various immune cells and stromal cells. It promotes the production of multiple AD-related inflammatory cytokines. TSLP upregulation is a hallmark of AD pathogenesis. To further elucidate the molecular action of chrysin in AD with respect to therapeutic efficacy, we focused on the effect exerted by chrysin on TSLP suppression in TNFα-stimulated keratinocytes and in a clinically relevant animal model with AD-like skin lesions induced upon DNCB challenge. We showed that chrysin suppresses TSLP expression by inhibiting the MAPKs ERK1/2 and JNK1/2 pathways and downregulating EGR1 expression in the inflammatory environment.

Various transcription factors, such as vitamin D3 receptor, NF-κB, and AP1 [43,44,52], are involved in the transcriptional regulation of *TSLP* based on the stimuli applied. To identify the *cis*-acting element responsible for mediating the effects of chrysin, we established a series of *TSLP* promoter–reporter constructs and evaluated the effect of chrysin on *TSLP* promoter activity in a luciferase activity assay. Cells transfected with the shortest reporter construct (−369/+18) continued to exhibit chrysin activity, suggesting that the chrysin response element is between the positions −369 and +18. We found a putative EGR1-binding motif, EBS, between the positions −206/−187 in the −369/+18 construct. *EGR1*, also named zinc finger protein 225 (*Zif268*), *Drosophila* Kr finger probe 24 (*Krox24*), tetradecanoyl phorbol acetate-induced sequence 8 (TIS8), and nerve growth factor-induced clone A (*NGFI-A*), is an immediate-early response gene that encodes a transcription factor containing three Cys2-His2-type zinc finger DNA-binding domains [53]. It regulates various cellular pathophysiological responses, including inflammation, synaptic plasticity, female reproduction, cell proliferation, apoptosis, and carcinogenesis [54,55,56,57,58,59]. 

EGR1 is expressed at high levels in damaged skin tissues [60]. EGR1 regulates the expression of genes encoding inflammation-related proteins, such as IL-33-induced TSLP [45], IL-17-induced psoriasin [61], and IL-13-induced kallikrein-related peptidase 7 (KLK7) in keratinocytes [62]. Recently, we demonstrated that immune cell infiltration in AD-like skin lesions was substantially attenuated in *Egr1*-knockout mice, and the TNFα-induced expression of cytokines, including TSLP, IL-1β, IL-6, CXCL1, CCL2, and CCL5, was inhibited in response to EGR1 knockdown [42]. Furthermore, the AB1711 compound, a small-molecule inhibitor targeting the EGR1 zinc-finger DNA-binding domains, was shown to abrogate the expression of EGR1-regulated inflammatory cytokines in keratinocytes and improve both skin inflammation and itching in DNCB-challenged NC/Nga mice [42]. These findings suggest that the inhibition of EGR1 transcriptional activity is a promising therapeutic strategy for improving therapeutic efficacy in chronic skin inflammation. In this study, the functional importance of the EBS within the *TSLP* promoter was analyzed via transient transfection experiments. We observed that the transient transfection of EGR1 enhanced the promoter-reporter activity of the −369/+18 construct. We further investigated whether chrysin inhibits EGR1 to downregulate TSLP expression.

We found that chrysin prevented TNFα-induced EGR1 DNA-binding activity, as observed using EMSA. In addition, chrysin inhibited TNFα-induced EGR1 expression in HaCaT keratinocytes. We also confirmed that the oral administration of chrysin attenuated both EGR1 and TSLP expression in vivo in the AD-like skin lesions of DNCB-challenged mice. These findings suggest that chrysin downregulates EGR1 expression to inhibit TSLP expression in the inflammatory microenvironment. One of the best-characterized transcription factors that regulate EGR1 expression is the Ets-like protein-1 (ELK-1), which is phosphorylated and activated by ERK1/2, p38 kinase, and JNK1/2 in response to mitogens and TNFα [63]. Our data showed that chrysin inhibited the TNFα-induced phosphorylation of ERK1/2 and JNK1/2 but not of p38 kinase, suggesting that chrysin downregulates EGR1 expression by differentially inhibiting the MAPK signaling pathways in HaCaT keratinocytes. 

NF-κB is a transcription factor expressed ubiquitously in almost all tissues, including skin keratinocytes. TNFα strongly activates the NF-κB signaling pathway in HaCaT keratinocytes [64]. NF-κB mediates TNFα-induced TSLP expression in human airway smooth muscle cells [43] and IL-1β-induced TSLP expression in intestinal epithelial cells [44]. We have previously reported that chrysin inhibits NF-κB activity by targeting the inhibitor of κB kinase, a protein encoded upstream of NF-κB and is involved in the proteolysis of the NF-κB inhibitor IκB [39]. Choi et al. [37] also reported that chrysin inhibits TNFα/IFNγ-induced degradation of IκB, leading to the inhibition of nuclear translocation of NF-κB in HaCaT keratinocytes. Therefore, chrysin may downregulate TSLP expression by inhibiting both EGR1 and NF-κB. 

## 4. Materials and Methods

### 4.1. Materials

Chrysin, DNCB, TB, and H&E staining kits were purchased from Sigma-Aldrich (St. Louis, MO, USA). TNFα was purchased from ProSpec-Tany TechnoGene, Ltd. (Ness-Ziona, Israel). A firefly luciferase assay system was obtained from Promega (Madison, WI, USA). Anti-TSLP antibody was obtained from Novus Biologicals (Centennial, CO, USA), and phospho-ERK1/2 (Thr202/Tyr204), phospho-p38 (Thr180/Tyr182), and phospho-JNK1/2 (Thr183/Tyr185) antibodies were obtained from Cell Signaling Technology (Danvers, MA, USA). Anti-GAPDH and anti-EGR1 antibodies were purchased from Santa Cruz Biotechnology (Dallas, TX, USA). A secondary antibody conjugated to rhodamine red-X was obtained from Jackson ImmunoResearch Laboratories (West Grove, PA, USA). 

### 4.2. Cells and Cell Culture

Human keratinocyte HaCaT cells were obtained from the Cell Line Service (Eppelheim, Germany). The cells were cultured in Dulbecco’s modified Eagle’s medium supplemented with 10% fetal bovine serum (HyClone, Logan, UT, USA) and penicillin-streptomycin (Sigma-Aldrich).

### 4.3. RT-PCR

Total RNA was isolated from the HaCaT cells using a TRIzol RNA Extraction Kit (Invitrogen, Carlsbad, CA, USA), and cDNA was synthesized using an iScript cDNA Synthesis Kit (Bio-Rad, Hercules, CA, USA). RT-PCR was performed using reverse transcriptase (Promega) and gene-specific PCR primers. The PCR primers used in this study were as follows:

- EGR1 forward, 5′-CAG CAG TCC CAT TTA CTC AG-3′;
- EGR1 reverse, 5′-GAC TGG TAG CTG GTA TTG-3;
- TSLP forward, 5′-TAG CAA TCG GCC ACA TTG CCT-3′;
- TSLP reverse, 5′-GAA GCG ACG CCA CAA TCC TTG-3;
- GAPDH forward, 5′-CCA AGG AGT AAG AAA CCC TGG AC-3′;
- GAPDH reverse, 5′-GGG CCG AGT TGG GAT AGG G-3′.

The thermal cycling conditions were as follows: denaturation at 94 °C for 5 min, followed by 30 cycles of denaturation at 94 °C for 30 s, annealing at 58 °C for 30 s, and elongation at 72 °C for 1 min. The amplified PCR products were separated by electrophoresis in a 2% agarose gel containing ethidium bromide and visualized under UV transillumination.

### 4.4. Quantitative Real-Time PCR (Q-PCR)

The mRNA levels of the genes were quantified using an iCycler iQ system with an iQ SYBR Green Supermix kit (Bio-Rad). Validated Q-PCR primers and SYBR Green-based fluorescent probes specific for *TSLP* (id: qHsaCIP0030468), *EGR1* (qHsaCEP0039196), and *GAPDH* (id: qHsaCEP0041396) were obtained from Bio-Rad. The thermal cycling conditions used for PCR were as follows: denaturation at 95 °C for 2 min, followed by 40 cycles of denaturation at 95 °C for 10 s and 60 °C for 45 s. The relative mRNA levels of *TSLP* or *EGR1* were normalized to those of *GAPDH* using the software provided by the manufacturer.

### 4.5. Western Blot Analysis

HaCaT cells were lysed in ice-cold cell lysis buffer supplemented with 50 mM Tris-HCl (pH 7.4), 1% NP-40, 0.25% Na-deoxycholate, 500 mM NaCl, 1 mM EDTA, 1 mM Na_3_VO_4_, 1 mM NaF, 10 μg/mL leupeptin, and 1 mM PMSF. The proteins were separated by electrophoresis in a 10% SDS-polyacrylamide gel and transferred to nitrocellulose membranes. After treatment with the appropriate primary and secondary antibodies, the blots were developed and observed using an enhanced chemiluminescence detection system (GE Healthcare, Piscataway, NJ, USA).

### 4.6. Construction of Human TSLP Promoter-Reporter Constructs

A *TSLP* promoter fragment spanning nucleotides −1384 to +18 upstream of the transcription start site was synthesized from human genomic DNA (Promega) via PCR using the primers 5′-CGT CCA ACC TCC TTT CTC CG -3′ (forward −1384F) and 5′-TTG GAG TCT CCC TGA TGC TCC AG-3′ (reverse, +18R). The amplified PCR products were ligated to a T&A vector (RBC Bioscience, Taipei County, Taiwan) and digested using *Kpn*I and *Hind*III. The products were ligated at the *Kpn*I and *Hind*III sites of the pGL4-basic vector (Promega), yielding pTSLP-Luc(−1384/+18). Several deletion constructs of the human *TSLP* promoter fragments were synthesized using PCR, for which the pTSLP-Luc(−1384/+18) construct was used as the template. The forward primer sequences were as follows:

−1338F: 5′-GGA CCA GAG CGA TGC AGG-3′
−1214F: 5′-CAT GAG CCA AGC CAG GGA G-3′
−1017F: 5′-AAA TCT GAG CCC GCC ATC TC-3′
−369F: 5′-GGG ACA TAT GCA AGG ACT CC-3′

One reverse primer, +18R, was used to generate the deletion constructs. The amplified PCR products were ligated to the T&A vector and then to the pGL4-basic vector. The insert sequence of each construct was confirmed using DNA sequencing (Macrogen, Seoul, Korea).

### 4.7. Luciferase Promoter–Reporter Assay

HaCaT keratinocytes cultured in 12-well plates were transfected with 0.2 µg of each *TSLP* promoter–reporter construct using Lipofectamine™ 2000 (Invitrogen) according to the manufacturer’s instruction. After 48 h of transfection, the cells were treated with TNFα in the presence or absence of chrysin (20 or 40 μM). After 8–12 h, the cells were harvested, and the firefly luciferase activity was measured using the Dual-Glo™ Luciferase assay system (Promega) following the manufacturer’s instruction. The relative luciferase activity of the untreated cells was assigned the value 1. Luminescence was measured using a dual luminometer (Centro LB960; Berthold Tech, Bad Wildbad, Germany).

### 4.8. EMSA

EMSA was performed using a LightShift Chemiluminescence EMSA kit (Thermo Fisher Scientific, Waltham, MA, USA), according to the manufacturer’s instruction. A biotin-labeled deoxyoligonucleotide probe corresponding to the EBS (5’-CAA AAA GGA GGA AGG TGA GGG AA-biotin-3’) was synthesized by Macrogen. Nuclear extracts (3 μg samples) prepared from the HaCaT keratinocytes were mixed with 50 fmole biotin-labeled EGR1-binding oligonucleotide probes with 1 µg poly(dI-dC) (Amersham Biosciences, Piscataway, NJ, USA). For the competition assay, 2.5 pmol of the unlabeled EGR1-binding oligonucleotide was added. DNA–protein complexes were separated in non-denaturing 6% polyacrylamide gels, and the antibody-reactive bands were visualized using chemiluminescence, according to the manufacturer’s instructions.

### 4.9. Induction of AD-Like Skin Lesions in the Ear and Neck of Mice

BALB/c mice (7-week-old, male) were obtained from Orient Bio, Inc. (Seongnam, Korea). The mice were housed in a specific pathogen-free environment at 20 ± 2 °C and a relative humidity of 50% ± 10%. The mice were randomly divided into three groups (based on the treatment administered): Group I, naive; Group II, DNCB + vehicle; and Group III, DNCB + chrysin (*n* = 5 in each group). In addiion to those in the naive group, all mice were sensitized with 4% SDS on both the neck and ear skin to disrupt the skin barrier; after 4 h, the SDS-sensitized areas were challenged with 1% DNCB dissolved in an acetone:olive oil mixture (1:3, v/v). The DNCB challenge was repeated once daily for 3 days. After a 4-day break, sensitization with 4% SDS followed by the application of 0.5% DNCB was repeated five times per week for 2 weeks (days 8–21). Chrysin powder was dissolved in dimethyl sulfoxide (250 mg/mL) to prepare a stock solution and then diluted using PBS to a final concentration of 25 mg/mL. The mice in Group III were administered chrysin (25 mg/kg) orally from day 7 (once daily, five times per week for 2 weeks). On day 22, all mice were euthanized, and tissue sections were prepared. The animal experiments were conducted in accordance with the guidelines for animal experiments and procedures approved by the Konkuk University Institutional Animal Care and Use Committee (IACUC). All experimental methods were confirmed to be in accordance with the relevant guidelines and regulations (approval number KU19129).

### 4.10. Histological Analysis

Skin sections of the neck and ear with AD-like lesions were fixed in 100% acetone and embedded in paraffin. Each section was cut (5 μm) using a microtome (Leica Microsystems, Wetzlar, Germany). The paraffin-embedded ear and neck skin sections were deparaffinized by treating with xylene (three times for 10 min) and hydrated using a graded ethanol series. After deparaffinization and rehydration, the tissue sections were stained with H&E. The infiltrated mast cells were stained with 0.1% TB. Images of each section were captured using a light microscope (EVOS FL Auto, Bothell, WA, USA). 

### 4.11. Immunohistochemical and Immunofluorescence Analysis

Immunostaining of the skin sections from the ear and neck was performed as previously described [39]. Briefly, after deparaffinization with xylene (three times for 10 min) and hydration with a graded ethanol series, the tissue sections were placed in 1 mM EDTA (pH 8.0) at 70 °C for 20 min. After rinsing with PBS, the tissue sections were incubated with 7% goat serum for 1 h to block non-specific binding of immunoglobulin (Ig). For EGR1 immunostaining, the sections were treated overnight with primary rabbit anti-EGR1 antibodies (1:100 dilution) at 4 °C. After washing three times with PBS, the sections were treated with biotinylated goat anti-rabbit IgG (1:100 dilution) at 25 °C for 1 h. Immunoreactivity was visualized after treatment with a diaminobenzidine substrate for 5 min, followed by counterstaining with H&E. 

For TSLP immunofluorescence staining, the tissue sections were treated overnight with an anti-TSLP antibody (1:100 dilution) at 4 °C. After washing, the cells were treated with a rhodamine red-X-conjugated secondary antibody (1:300 dilution) at 25 °C for 1 h. The nuclei were counterstained with Hoechst 33258 solution for 10 min. After extensive washing with PBS, the slides were mounted using the ProLong Gold Antifade reagent (Invitrogen). Fluorescent images were captured using an EVOS FL fluorescence microscope (Advanced Microscopy Group; Bothell, WA, USA). 

### 4.12. Statistical Analysis

Data are expressed as mean ± standard deviation. Statistical analysis was performed using one-way analysis of variance, followed by Dunnett’s or Sidak’s multiple comparisons test using GraphPad Prism (version 9.0.1; GraphPad Software, Inc., La Jolla, CA, USA). Statistical significance was set at *p* < 0.05.

## 5. Conclusions

To the best of our knowledge, this is the first study to demonstrate that chrysin suppresses *TSLP* expression by downregulating ERK1/2- and JNK1/2-dependent EGR1 expression in the skin inflammatory microenvironment. We believe that the results of this study will improve our understanding of the mode of action of chrysin and its therapeutic efficacy in AD.

## Data Availability

The data presented in this study are available on request from the corresponding author.

## Abbreviations

AD	atopic dermatitis
CCL	C-C motif chemokine ligand
DNCB	2,4-dinirochlorobenzene
EBS	EGR1-binding sequence
EGR1	early rrowth response 1
shEgr1	EGR1 shRNA
EMSA	electrophoretic mobility shift assay
ERK	Extracellular signal-regulated kinases
GAPDH	glyceraldehyde 3-phosphate dehydrogenase
H&E	hematoxylin and eosin
Ig	immunoglobulin
IKK	inhibitor of κB kinase
IL	interleukin
JNK	c-Jun N-terminal kinase
MAPK	mitogen-activated protein kinases
NF-κB	nuclear factor-κB
Q-PCR	quantitative real-time PCR
RT-PCR	reverse-transcription polymerase chain reaction
shCT	scrambled shRNA
TB	toluidine blue
Th2	T helper cell 2
TNFα	tumor necrosis factor-alpha
TSLP	thymic stromal lymphopoietin
